# Cancer mortality patterns in selected Northern and Southern African countries

**DOI:** 10.1097/CEJ.0000000000000852

**Published:** 2023-11-24

**Authors:** Margherita Pizzato, Claudia Santucci, Fabio Parazzini, Eva Negri, Carlo La Vecchia

**Affiliations:** a Department of Clinical Sciences and Community Health, University of Milan, Milan; b Department of Medical and Surgical Sciences, University of Bologna, Bologna, Italy

**Keywords:** Africa, cancer mortality, joinpoint analysis, Northern African countries, Southern African countries

## Abstract

**Background:**

Non-communicable diseases have been rapidly increasing in African countries. We provided updated cancer death patterns in selected African countries over the last two decades.

**Methods:**

We extracted official death certifications and population data from the WHO and the United Nations Population Division databases. We computed country- and sex-specific age-standardized mortality rates per 100 000 person-years for all cancers combined and ten major cancer sites for the periods 2005–2007 and 2015–2017.

**Results:**

Lung cancer ranked first for male cancer mortality in all selected countries in the last available period (with the highest rates in Réunion 24/100 000), except for South Africa where prostate cancer was the leading cause of death (23/100 000). Prostate cancer ranked second in Morocco and Tunisia and third in Mauritius and Réunion. Among Egyptian men, leukemia ranked second (with a stable rate of 4.2/100 000) and bladder cancer third (3.5/100 000). Among women, the leading cancer-related cause of death was breast cancer in all selected countries (with the highest rates in Mauritius 19.6/100 000 in 2015–2017), except for South Africa where uterus cancer ranked first (17/100 000). In the second rank there were colorectal cancer in Tunisia (2/100 000), Réunion (9/100 000) and Mauritius (8/100 000), and leukemia in Egypt (3.2/100 000). Colorectal and pancreas cancer mortality rates increased, while stomach cancer mortality rates declined.

**Conclusion:**

Certified cancer mortality rates are low on a global scale. However, mortality rates from selected screening detectable cancers, as well as from infection-related cancers, are comparatively high, calling for improvements in prevention strategies.

## Introduction

The burden of cancer has been growing in Africa due to population aging and growth, as well as to the increased prevalence of risk factors associated with the economic transition (Mayosi *et al.*, 2009; Parkin *et al.*, 2014). However, infrastructures for cancer management are limited, struggling to meet the rising cancer demand (Znaor *et al*., 2018). In addition, epidemiologic data are scant, due to death certification and cancer registration systems of inadequate quality and coverage (Zanetti *et al.*, 2010; Kantelhardt *et al*., 2015).

Over the last decades, we published cancer mortality assessments for selected European, Latin American and Australasian countries based on the WHO database (Santucci *et al*., 2023; Malvezzi *et al*., 2023; Pizzato *et al*., 2022), while we have not provided corresponding figures for the African continent. Despite some inherent uncertainties in the African estimates, the calculation of mortality rates, along with their trends, is useful for risk factors monitoring and health planning.

We here consider cancer mortality patterns in selected African countries, using data from the WHO. We included three Northern African countries (i.e. the Arab Republic of Egypt, the Kingdom of Morocco, the Republic of Tunisia), the Republic of South Africa, as well as two Southern African Islands (i.e. the Republic of Mauritius, and Réunion).

## Material and methods

We extracted official death certifications and population data from the WHO and the United Nations Population Division databases for all available calendar years starting from 1990 (United Nations DoEaSA, 2017; World Health Organization Statistical Information System, 2022). We computed country- and sex-specific age-standardized mortality rates (ASMRs) using the world standard population per 100 000 persons at all ages for all cancers combined and for 10 major cancer sites (see Supplementary Table 1, Supplemental digital content 1, http://links.lww.com/EJCP/A417 for the list of cancers and the corresponding International Classification Codes used). We reported rates for the periods 2005–2007 and 2015–2017, along with the corresponding percent changes. We carried out a joinpoint regression analysis on mortality data for all cancers combined over the whole available period for each country except for Tunisia where only a few calendar years were available (Joinpoint Regression Program April, 2022). We thus identified the time point(s), called ‘joinpoints’, when a change in the linear slope of the temporal trend occurred, by testing up to a maximum of four joinpoints. As a summary measure, we showed the estimated annual percent change (APC) for each identified linear segment, and the weighted average APC (AAPC) over the entire available study period (Kim *et al.*, 2000; Clegg *et al.*, 2009).

For analyses, we used the software R version 4.2.2 (R Development Core Team, 2017), SAS version 9.4 (SAS Institute Inc., Cary, NC, USA), and Joinpoint Regression Program version 4.9.1.0.

## Results

**Fig. 1:**
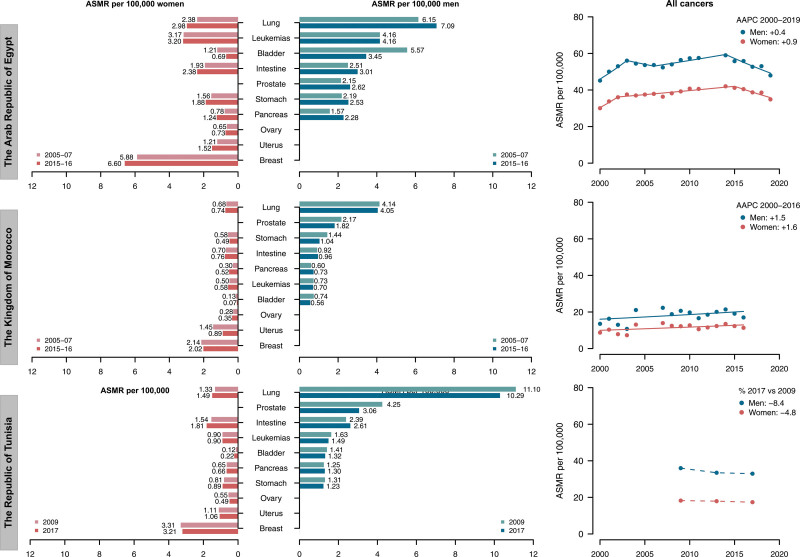
Age-standardized mortality rates (ASMRs) for major cancer sites in the Arab Republic of Egypt, the Kingdom of Morocco, and the Republic of Tunisia, along with the trends in ASMRs (dots) from all cancers and the corresponding joinpoint models (lines) over the available period.

Table 1 shows the ASMRs in 2005–2007 and 2015–2017 with the average annual certified deaths in 2015–2017, along with the percent change between the two periods in the Arab Republic of Egypt, the Kingdom of Morocco, and Tunisia. Corresponding data along with the trends in ASMRs (dots) from all cancers and the corresponding joinpoint models (lines) since 2000 are shown in Fig. 1. In Egypt, the most recent ASMR for all cancers was 55/100 000 for men and 40/100 000 for women. Corresponding values were 33/100 000 men and 17/100 000 women in Tunisia, and 18/100 000 men and 12/100 000 women in Morocco. Among men, lung cancer was the first ranking site in all selected countries, with the latest ASMRs of 10.3/100 000 in Tunisia, 7.1/100 000 in Egypt (+15% vs. 2005–2007), and 4.1/100 000 in Morocco. Prostate cancer was the second leading cause of cancer-related death both in Morocco (1.8/100 000 in 2015–16, −16% vs. 2005–2007) and Tunisia (3.1/100 000 in 2017, −28% vs. 2013), while in Egypt leukemia ranked second (with a stable ASMR of 4.2/100 000). Among women, ASMRs from breast cancer exceed those from any other sites; the most recent rates were 6.6/100 000 in Egypt (+12% vs. 2005–2007), 3.2/100 000 in Tunisia (−3% vs. 2009), and 2.0/100 000 in Morocco (−6% vs. 2005–2007). The second leading causes of cancer death were leukemia in Egypt (3.2/100 000), uterus in Morocco (0.9/100 000), and colorect colorectum in Tunisia (1.8/100 000).

**Table 1:** Age-standardized (world population) mortality rates and annual average deaths from selected cancer sites and all cancer combined per 100 000 person-years in 2005–2007 and 2015–2017 (unless indicated) for both sexes, along with the corresponding change in rates (Δ%) in the selected Northern African countries

Cancer site	Men				Women			
	ASMR 2005–2007^a^	Annual average deaths 2015–2017^b^	ASMR 2015–2017^b^	Δ% 2015–2017 vs. 2005–2007	ASMR 2005–2007^a^	Annual average deaths 2015–2017^b^	ASMR 2015–2017^b^	Δ% 2015–2017 vs. 2005–2007
The Arab Republic of Egypt								
Stomach	2.19	883	2.53	15.5	1.56	743	1.88	20.5
Colorectum	2.51	1064	3.01	19.9	1.93	942	2.38	23.3
Pancreas	1.57	777	2.28	45.2	0.78	474	1.24	59.0
Lung	6.15	2440	7.09	15.3	2.38	1169	2.98	25.2
Breast	0.17	52	0.15	−11.8	5.88	2573	6.60	12.2
Uterus	-	-	-	-	1.21	586	1.52	25.6
Ovary	-	-	-	-	0.65	284	0.73	12.3
Prostate	2.15	793	2.62	21.9	-	-	-	-
Bladder	5.57	1119	3.45	−38.1	1.21	270	0.69	−43.0
Leukemias	4.16	1660	4.16	0.0	3.17	1351	3.20	0.9
All cancers	53.20	19 110	54.72	2.9	37.23	15 823	40.10	7.7
The Kingdom of Morocco								
Stomach	1.44	185	1.04	−27.8	0.58	98	0.49	−15.5
Colorectum	0.92	172	0.96	4.3	0.70	151	0.76	8.6
Pancreas	0.60	129	0.73	21.7	0.30	101	0.52	73.3
Lung	4.14	751	4.05	−2.2	0.68	147	0.74	8.8
Breast	0.25	14	0.08	−68.0	2.14	395	2.02	−5.6
Uterus	-	-	-	-	1.45	179	0.89	−38.6
Ovary	-	-	-	-	0.28	71	0.35	25.0
Prostate	2.17	277	1.82	−16.1	-	-	-	-
Bladder	0.74	94	0.56	−24.3	0.13	15	0.07	−46.2
Leukemias	0.73	125	0.70	−4.1	0.50	106	0.58	16.0
All cancers	22.28	3173	18.01	−19.2	13.92	2319	11.80	−15.2
Tunisia								
Stomach	1.31	95	1.23	−6.1	0.81	75	0.89	9.9
Colorectum	2.39	205	2.61	9.2	1.54	165	1.81	17.5
Pancreas	1.25	100	1.30	4.0	0.65	57	0.66	1.5
Lung	11.10	753	10.29	−7.3	1.33	134	1.49	12.0
Breast	0.09	17	0.17	88.9	3.31	263	3.21	−3.0
Uterus	-	-	-	-	1.11	84	1.06	−4.5
Ovary	-	-	-	-	0.55	37	0.49	−10.9
Prostate	4.25	236	3.06	−28.0	-	-	-	-
Bladder	1.41	97	1.32	−6.4	0.12	22	0.22	83.3
Leukemias	1.63	112	1.49	−8.6	0.90	72	0.90	0.0
All cancers	35.98	2473	32.95	−8.4	18.24	1462	17.37	−4.8

ASMR, age-standardized mortality rate.

a 2009 for Tunisia.

b 2015–2016 for The Arab Republic of Egypt and The Kingdom of Morocco and 2017 for Tunisia.

The trends in mortality from major cancer sites in Egypt and Morocco are reported in Supplementary Figures 1 and 2, Supplemental digital content 1, http://links.lww.com/EJCP/A417 while the corresponding results of the joinpoint regression analysis are reported in Supplementary Table 2, Supplemental digital content 1, http://links.lww.com/EJCP/A417. During the whole period 2000–2019 in Egypt, we observed a significant increase in rates in both sexes from stomach cancer (AAPC: +1.1% in men and +1.6% in women), lung cancer (+1.8% in men and +2.9% in women), and among women from pancreatic cancer (+2.7%). Mortality from bladder cancer showed favorable patterns in both sexes (AAPC: −5.2% in men and −4.1% in women). Among Moroccan men, during the period 2000–2016, no favorable patterns were observed. We registered significant AAPC of +3.7% for colorectum, +7.3% for pancreas, and +4.3% for lung cancer. In the same calendar years, women showed significant increases in mortality trend from cancer of colorectum (AAPC: +4%), pancreas (+7.5%), lung (+5.4%), breast (+6.4%), uterus (+4%), and leukemias (+3.9%).

**Fig. 2:**
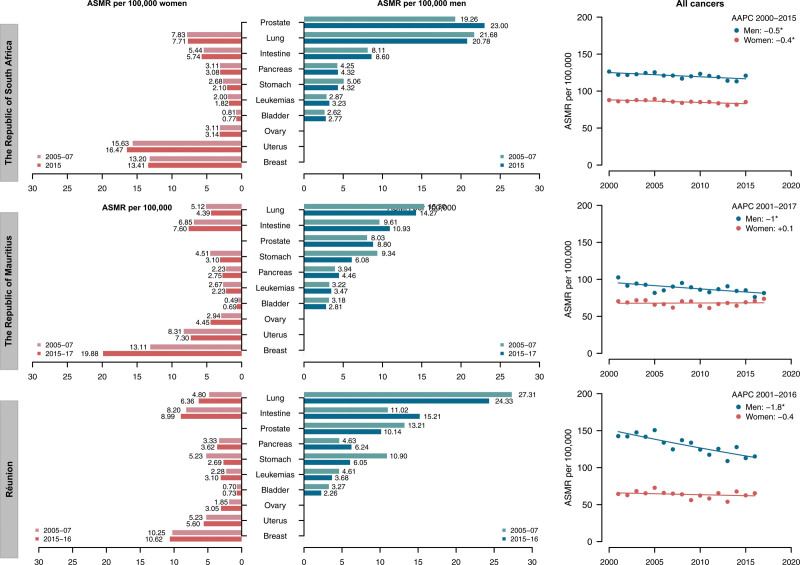
Age-standardized mortality rates (ASMRs) for major cancer sites in the Republic of South Africa, the Republic of Mauritius and Réunion, and the trends in ASMRs (dots) from all cancers and the corresponding joinpoint models (lines) over the available period.

Table 2 reports the ASMRs in 2005–2007 and 2015–2017 with the average annual deaths in 2015–2017, along with the percent change between the two periods in the Republic of South Africa, the Republic of Mauritius, and Réunion. Corresponding data along with the trends in ASMRs (dots) from all cancers and the corresponding joinpoint models (lines) since 2000 are shown in Fig. 2. Mortality trends for all cancers have been favorable since 2000 for all selected countries in both sexes. Lung cancer, albeit generally declining, remained the leading cause of male cancer death in Réunion (24.3/100 000 in 2015–16, −11% vs. 2005–2007) and Mauritius (14.3/100 000 in 2015–2017, −7% vs. 2005–2007), and the second one in South Africa (20.8/100 000 in 2015, −4% vs. 2005–2007). Prostate cancer was the first ranking site in South Africa (23/100 000 in 2015, + 19% vs. 2005–2007), while it ranked third in Réunion and Mauritius (10.1/100 000 and 8.8/100 000 respectively). Among women, the leading cancer cause of death was breast in Mauritius (19.9/100 000 in 2015–2017, +52% vs. 2005–2007) and Réunion (10.6/100 000 in 2015–16, +4% vs. 2005–2007), uterus in South Africa (16.5/100 000 in 2015, +5% vs. 2005–2007). Colorectal cancer rates increased in both sexes, reaching ASMRs of 15.2/100 000 men and 9/100 000 women in Réunion, and 10.0/100 000 men and 7.6/100 000 women in Mauritius. Stomach cancer mortality declined in all the three selected countries and both sexes. ASMRs from ovarian cancer increased both in Réunion (+65%) and Mauritius (+51%).

**Table 2:** Age-standardized (world population) mortality rates and annual average deaths from selected cancer sites and all cancer combined per 100 000 person-years in 2005–2007 and 2015–2017 (unless indicated) for both sexes, along with the corresponding change in rates (Δ%) in the selected Southern African countries

Cancer site	Men				Women			
	ASMR 2005–2007	Annual average deaths 2015–2017^a^	ASMR 2015–2017^a^	Δ% 2015–2017 vs. 2005–2007	ASMR 2005–2007	Annual average deaths 2015–2017^a^	ASMR 2015–2017^a^	Δ% 2015–2017 vs. 2005–2007
The Republic of South Africa								
Stomach	5.06	749	4.32	−14.6	2.68	512	2.10	−21.6
Colorectum	8.11	1466	8.60	6.0	5.44	1377	5.74	5.5
Pancreas	4.25	750	4.32	1.6	3.11	725	3.08	−1.0
Lung	21.68	3746	20.78	−4.2	7.83	1852	7.71	−1.5
Breast	0.42	57	0.36	−14.3	13.20	3297	13.41	1.6
Uterus	-	-	-	-	15.63	4171	16.47	5.4
Ovary	-	-	-	-	3.11	776	3.14	1.0
Prostate	19.26	3033	23.00	19.4	-	-	-	-
Bladder	2.62	415	2.77	5.7	0.81	179	0.77	−4.9
Leukemias	2.87	609	3.23	12.5	2.00	447	1.82	−9.0
All cancers	122.32	20 710	120.82	−1.2	87.34	21 032	85.23	−2.4
The Republic of Mauritius								
Stomach	9.34	48	6.08	−34.9	4.51	32	3.10	−31.3
Colorectum	9.61	88	10.93	13.7	6.85	78	7.60	10.9
Pancreas	3.94	36	4.46	13.2	2.23	28	2.75	23.3
Lung	15.30	115	14.27	−6.7	5.12	44	4.39	−14.3
Breast	0.66	1	0.22	−66.7	13.11	188	19.88	51.6
Uterus	-	-	-	-	8.31	72	7.30	−12.2
Ovary	-	-	-	-	2.94	41	4.45	51.4
Prostate	8.03	68	8.80	9.6	-	-	-	-
Bladder	3.18	22	2.81	−11.6	0.49	7	0.69	40.8
Leukemias	3.22	26	3.47	7.8	2.67	20	2.23	−16.5
All cancers	85.72	644	80.86	−5.7	64.62	690	71.30	10.3
Réunion								
Stomach	10.90	37	6.05	−44.5	5.23	23	2.69	−48.6
Colorectum	11.02	95	15.21	38.0	8.20	67	8.99	9.6
Pancreas	4.63	39	6.24	34.8	3.33	28	3.62	8.7
Lung	27.31	149	24.33	−10.9	4.80	47	6.36	32.5
Breast	0.55	1	0.16	−70.9	10.25	75	10.62	3.6
Uterus	-	-	-	-	5.23	41	5.60	7.1
Ovary	-	-	-	-	1.85	24	3.05	64.9
Prostate	13.21	64	10.14	−23.2	-	-	-	-
Bladder	3.27	15	2.26	−30.9	0.70	7	0.73	4.3
Leukemias	4.61	23	3.68	−20.2	2.28	23	3.10	36.0
All cancers	135.94	702	114.04	−16.1	67.67	487	63.96	−5.5

ASMR, age-standardized mortality rate.

a 2015 for The Republic of South Africa and 2015–2016 for Réunion.

The trends in mortality from major cancer sites in South Africa, Mauritius and Réunion are reported in Supplementary Figures 3–5, Supplemental digital content 1, http://links.lww.com/EJCP/A417 while the corresponding results of the joinpoint regression analysis are reported in Supplementary Table 3, Supplemental digital content 1, http://links.lww.com/EJCP/A417. During the available period (i.e. 1993–2019) in South Africa, we observed a significant decrease in rates in both sexes from stomach cancer (AAPC: −2.8% in men and −3.6% in women), lung cancer among men (−0.8%), and bladder cancer among women (−1.1%). Significant unfavorable mortality patterns were reported among males for colorectal (+1.2%), pancreatic (+0.6%), prostate cancer (+2.3%), and leukemia (+0.6%) as well as among females for breast (+1%) and ovarian cancer (+2%). In Mauritius, during the period 2001–2017, we registered a significant increase for both sexes in mortality trend for colorectal cancer (AAPC was +1.3% in male and +1.5% in female), pancreas (+2% in male and +1.9% in female), and breast (+2%) and ovarian cancer (+3.3%) among women. Decreasing pattern in mortality was observed in both sexes for stomach cancer (AAPC was −4.1% in male and −4% in female), among men for lung cancer (−1.7%), and among women for uterus (−1.8%). In Réunion, during the period 2001–2016, stomach cancer showed a decrease in rates for both sexes (AAPC: −4% among men and −3.1% among women). Significant downward trends were registered among men for leukemias (AAPC: −3%), lung (−1.3%) and prostate (−2.6%) cancers. Women showed a significant increase in trend for lung cancer (AAPC: +4%), while the other cancer sites showed stable mortality rates over the analyzed years.

## Discussion

Certified mortality rates for *all cancers combined* were mostly stable or favorable in the selected African countries over recent decades. The leading causes of cancer-related death were lung, prostate and colorectal cancers in men, breast, uterus, and colorectal cancers in women.

*Breast cancer* has been rising in many transitioning countries, representing to date a leading cause of cancer mortality (about 1 in every 6 female cancer deaths in the selected African countries) (Arnold *et al*., 2022). Changes in reproductive behaviors (e.g. delayed age at first pregnancy, lower parity, and shorter duration of breastfeeding), as well as in lifestyle factors (e.g. higher alcohol consumption and physical inactivity) exposed women from these areas to a higher breast cancer risk (Corbex *et al.*, 2014; Joko-Fru *et al*., 2020). Further, as compared to other ethnicities, hormone receptor-negative breast cancers, the histotypes characterized by early onset and aggressive behavior are more frequent in Black women (Chouchane *et al.*, 2013; Lukong *et al.*, 2017). Despite sporadic efforts have been devoted over the last decade to implement screening and early diagnosis programs, to ensure broader access to high-quality health services, and raise breast cancer awareness (Stefan *et al*., 2013; Basu *et al*., 2018; Mrabti *et al*., 2021), African women still face relevant delays in breast cancer detection, as well as limited access to affordable treatments (Harford, 2011; DeSantis *et al.*, 2015).

Differences in smoking patterns explained most of the variation in *lung cancer* rates across geography and time (Cheng *et al.*, 2016). In the selected countries, smoking prevalence, albeit declining over the last decades, remained notably high among men (especially in Egypt, Tunisia and Mauritius), while are comparatively low among women (especially in the Arab countries) (Azagba *et al.*, 2015; Jazieh *et al*., 2019). Decreased exposure to occupational carcinogens, as well as to indoor and outdoor pollution, may have had a role in the decline of lung cancer rates (Bello *et al.*, 2011). Chronic Pulmonary Infections namely tuberculosis (whose reported prevalence in South Africa remained one of the highest worldwide), may contribute to the lung cancer burden, even in non-smokers (Corrales *et al.*, 2020).

Favorable *stomach cancer* mortality trends can be attributed to improvements in the control of *Helicobacter pylori* (Hp) infection, advancements in food preservation and a better and more varied diet (Ogundipe *et al.*, 2018; Shokouhi *et al.*, 2021). As compared to the selected Southern African countries (whose ASMRs were in line with those observed in Europe), stomach cancer mortality rates were lower in the Northern African countries. In these latter areas, beyond possible under-reporting, and despite historically high levels of Hp infection, genetic and environmental (dietary) factors could act as possible protective determinants (Elghali *et al*., 2018).

Unfavorable changes in identified risk factors (e.g. increased sedentariness, high-fat dietary patterns) at times not counterbalanced by broad access to high-quality health services, may explain *pancreas and colorectum cancer* increasing trends (Katsidzira *et al.*, 2017). The prevalence of overweight has been also rising in the selected African countries reaching to date levels similar to those observed in high-income countries, especially among women (Finucane *et al*., 2011; Selmouni *et al*., 2018). Except for Réunion, where screening services retraced those implemented in France, other selected countries lacked early detection programs for colorectal cancer (Laiyemo *et al.*, 2016). Moreover, the lower alcohol consumption reported among both sexes in the Arab countries, as compared to the Southern African is, represented a favorable factor in these areas (Bryazka *et al*., 2022).

*Prostate* cancer mortality rates were over two times higher in South Africa than in Réunion and Mauritius (whose rates were in line with the European ones) (Culp *et al.*, 2020). Black African ancestry was associated with higher prostate cancer mortality rates, which, at least in part, is thought to reflect a genetic susceptibility to develop prostate malignancies characterized by aggressive biology and unfavorable anatomical localization (Adeloye *et al*., 2016; Seraphin *et al*., 2021). Disparities in prostate cancer mortality rates also reflected geographic differences in the availability of facilities for the detection of early-stage lesions and to adequate management and treatment (McGinley *et al.*, 2016).

While *cervical cancer* deaths have been declining in several high-income countries over recent decades, the its burden remained extremely high in Africa countries, reaching 20% of female cancer-related deaths in South Africa (Jedy-Agba *et al*., 2020). Even in those African countries where programs for cervical cancer prevention have been implemented over the last decades, the national coverage of both cervical screening and HPV vaccination, as well as the attendance to second-line diagnosis and management after positive screening results, remained comparatively low (Elmajjaoui *et al.*, 2016; Olorunfemi *et al.*, 2018). HIV infection, whose prevalence in South Africa is among the highest globally has been found to increase the rate of HPV infection and its persistence (Mboumba Bouassa *et al.*, 2017).

The westernization of lifestyle (including reduced parity and breastfeeding, late age at first pregnancy, and increase in obesity and type 2 diabetes) justify, at least in part, the *ovarian cancer* increases in death rates (Koon Sun Pat *et al.*, 2019; Zheng *et al*., 2020; Gizaw *et al*., 2023). Ovarian cancer clinical characteristics (i.e. insidious onset with nonspecific symptoms, early resistance to conventional treatment and high recurrence) hinder its clinical management in low-resource countries (Cabasag *et al*., 2022).

The eradication programs of schistosomiasis, through praziquantel mass drug administration and sanitation measures implemented in the selected Northern African countries over the last decades, explain the declining trends observed for *bladder cancer* (Adeloye *et al*., 2019; Parkin *et al.*, 2020). The male-to-female ratio (M: F) was in the Southern African countries in line with that observed in Europe (around 4 : 1); the wider gender gap seen in Northern African countries (up to 8 : 1 in Morocco) reflects the strong gender disparities in smoking consumption and possibly occupational exposure to aromatic amines (Felix *et al*., 2008; El Sayed *et al.*, 2018).

The high burden of *leukaemia and other lymphatic malignancies* in Egypt is likely driven by the spread of the Hepatitis C Virus (HCV), whose infection has been associated with haematological cancers (Goldman *et al*., 2009; Dey *et al*., 2011). The parenteral anti-Schistosoma mass campaign, which was carried out during the 1950s–80s before the praziquantel administration, established a very large reservoir of HCV in this country (Herzog *et al*., 2012; El Kassas *et al.*, 2018). Poor infection control, transfusion of unscreened blood, and inadequate sterilization procedures in medical and dental settings continue to fuel the epidemic, in particular in rural areas (Hussein *et al*., 2016).

African patients faced multiple barriers in seeking and accessing adequate healthcare services (Harford, 2015). Needing to travel long distances to obtain cancer care facilities, concentrated in urban centers, hindered effective cancer management. Financial constraints, lower levels of cancer symptom awareness, reliance on traditional healers, embarrassment towards a still stigmatized disease, and fatalistic beliefs about death also account for the delay in seeking diagnosis (Pourette *et al*., 2022). In addition, language barriers have a potential role in suboptimum management: while health providers may be fluent only in the official language, patients are better expressed in sublanguages and dialects (Hayes and Bornman, 2017). Health systems remained generally understaffed and underfinanced, especially in the public health care system, where patients are managed with the use of simpler techniques and basic therapies. Effective health care is also limited by the lack of trained medical personnel, insufficient modern equipment, poor infrastructure, and the high cost of cancer drugs, including targeted therapies that remain prohibitively expensive in most low-resource settings (Kingham *et al*., 2013; Vanderpuye *et al*., 2017). The facilities equipped and empowered to ensure prevention are struggling to develop; whether offered, prevention services are primarily opportunistic and out-of-pocket paid (Made *et al*., 2017). Suboptimum pathology, as well as poor availability of adjuvant radiotherapy and chemotherapy, resulted in the underuse of conservative surgery (Jemal *et al*., 2012; Adesina *et al*., 2013). Underrepresentation of certain ethnicities in cancer trials may account for worse survival rates in those population subgroups (Odedina *et al*., 2020).

Data quality remains a major limitation in understanding and estimating the mortality of the African continent (Pace and Shulman, 2016; Belbaraka *et al.*, 2022). Cancer registration and death certification faced multiple obstacles in these areas, including low resource allocation, inadequate health infrastructures and informatic systems, and lack of specialized technical staff (Bray *et al*., 2022). Given the modest coverage and the inaccuracy in death certification, especially in Northern African countries, reported mortality rates must be interpreted with caution (Elidrissi Errahhali *et al.*, 2017). The major strength of this study lies in the fact that it provides one of the few appraisals of mortality patterns across different areas of the African continent, based on a WHO data source.

### Conclusion

The wide African heterogeneity in terms of ethnic and genetic backgrounds, demographic characteristics, and economic status is reflected in the epidemiological patterns observed. Genetic characteristics in Africans modulate their risks to specific cancer types, particularly breast and prostate cancers, and influence the clinical manifestation of these malignancies. Some of the observed increases in rates likely reflect the adoption of transitional countries lifestyles in selected African areas. The burden of infection-related cancers still endured in all selected contexts. Coupling this with the lack of screening, treatment facilities and resources, a greater focus is needed on primary and secondary prevention.

## Acknowledgements

This work was supported by the Foundation of the Italian Association for Cancer Research (AIRC Foundation, project N. 22987). CS and CLV are also supported by EU funding within the NextGenerationEU-MUR PNRR Extended Partnership initiative (Project no. PE00000007, INF-ACT). The funding sources had no role in the design and conduct of the study; collection, management, analysis, and interpretation of the data; preparation, review, or approval of the manuscript; and the decision to submit the manuscript for publication.

The data that support the findings of this study are openly available in the WHO database at https://platform.who.int/mortality/themes/theme-details/topics/topic-details/MDB/malignant-neoplasms.

### Conflicts of interest

There are no conflicts of interest.

## Supplementary Material
