# Supplementary material for: Cancer mortality patterns in selected Northern and Southern African countries
Source: Eur J Cancer Prev. 2023 Nov 24;33(3):192–9. doi: 10.1097/CEJ.0000000000000852 (PMC10965137; doi:10.1097/CEJ.0000000000000852)

SUPPLEMENTARY MATERIAL

Supplementary Table 1. List of malignant neoplasms. 2

Supplementary Table 2. Joinpoint analysis of cancer mortality rates in men and women at all ages in the Arab Republic of Egypt (2000–2019) and the Kingdom of Morocco (2000–2016). 3

Supplementary Table 3. Joinpoint analysis of cancer mortality rates in men and women at all ages in in the Republic of South Africa (1993–2015), The Republic of Mauritius (2001–2017), and Réunion (2001–2016). 4

Supplementary Figure 1. Joinpoint analysis for mortality from major cancer sites in the Arab Republic of Egypt during the period 2000–2019. 6

Supplementary Figure 2. Joinpoint analysis for mortality from major cancer sites in the Kingdom of Morocco during the period 2000–2016. 7

Supplementary Figure 3. Joinpoint analysis for mortality from major cancer sites in the Republic of South Africa during the period 1993–2015 8

Supplementary Figure 4. Joinpoint analysis for mortality from major cancer sites in the Republic of Mauritius during the period 2001–2017 9

Supplementary Figure 5. Joinpoint analysis for mortality from major cancer sites in the Réunion during the period 2001–2016. 10

Supplementary Table 1. List of malignant neoplasms.

Neoplasm	ICD-10 code
Stomach	C16
Colorectum	C17-C21, C26
Pancreas	C25
Lung	C33-C34
Breast	C50
Uterus	C53-C55
Ovary	C56
Prostate	C61
Bladder	C67
Leukaemias	C91-C95
All cancers (malignant and benign)	C00-D48

ICD: International Classification of Diseases.

Supplementary Table 2. Joinpoint analysis of cancer mortality rates in men and women at all ages in the Arab Republic of Egypt (2000–2019) and the Kingdom of Morocco (2000–2016).

Cancer site	Men									Women								
	**Years1**	**APC1**	**Years2**	**APC2**	**Years3**	**APC3**	**Years4**	**APC4**	**AAPC**	**Years1**	**APC1**	**Years2**	**APC2**	**Years3**	**APC3**	**Years4**	**APC4**	**AAPC**
**The Arab Republic of Egypt**																		
Stomach	2000-2014	2.7*	2014-2019	-3.4*					1.1*	2000-2014	4*	2014-2019	-4.8*					1.6*
Colorectum	2000-2009	0.8	2009-2014	4.8	2014-2019	-2.5			1	2000-2002	15	2002-2009	0.3	2009-2014	5.2	2014-2019	-1.8	2.5
Pancreas	2000-2002	-11	2002-2008	1.8	2008-2014	6.4	2014-2019	-1.1	1	2000-2005	0.1	2005-2016	5.2*	2016-2019	-2			2.7*
Lung	2000-2011	3.9*	2011-2019	-1.1					1.8*	2000-2010	6.2*	2010-2019	-0.6					2.9*
Breast	-	-	-	-	-	-	-	-	-	2000-2015	1.6*	2015-2019	-3.3*					0.5
Uterus	-	-	-	-	-	-	-	-	-	2000-2017	2*	2017-2019	-21.8*					-0.8
Ovary	-	-	-	-	-	-	-	-	-	2000-2019	1							1
Prostate	2000-2015	2.4*	2015-2019	-4.8					0.9	-	-	-	-	-	-	-	-	-
Bladder	2000-2015	-3.7*	2015-2019	-10.8*					-5.2*	2000-2005	0.2	2005-2019	-5.6*					-4.1*
Leukemias	2000-2002	21.3*	2002-2016	0.1	2016-2019	-7.1*			0.9	2000-2002	24.8	2002-2015	0.7	2015-2019	-6.9*			1.3
All cancers	2000-2003	6.9*	2003-2006	-1.9	2006-2014	1.4	2014-2019	-3.7*	0.4	2000-2002	9.2	2002-2015	1.2*	2015-2019	-3.9*			0.9
**The Kingdom of Morocco**																		
Stomach	2000-2016	0							0	2000-2016	1.8							1.8
Colorectum	2000-2016	3.7*							3.7*	2000-2016	4*							4*
Pancreas	2000-2016	7.3*							7.3*	2000-2016	7.5*							7.5*
Lung	2000-2016	4.3*							4.3*	2000-2016	5.4*							5.4*
Breast	-	-	-	-	-	-	-	-	-	2000-2007	15.5*	2007-2016	-0.1					6.4*
Uterus	-	-	-	-	-	-	-	-	-	2000-2016	-1.1							-1.1
Ovary	-	-	-	-	-	-	-	-	-	2000-2016	4*							4*
Prostate	2000-2007	17	2007-2016	-0.6					6.8	-	-	-	-	-	-	-	-	-
Bladder	2000-2016	1.8							1.8	2000-2014	3.5*	2014-2016	-33.1					-2
Leukemias	2000-2016	1.8							1.8	2000-2016	3.9*							3.9*
All cancers	2000-2016	1.5							1.5	2000-2016	1.6							1.6

APC: annual percent change. AAPC: average annual percent change. *Significantly different from 0 ($p<0.05$).

Supplementary Table 3. Joinpoint analysis of cancer mortality rates in men and women at all ages in in the Republic of South Africa (1993–2015), The Republic of Mauritius (2001–2017), and Réunion (2001–2016).

Cancer site	Men					Women								
	Years1	APC1	Years2	APC2	AAPC	Years1	APC1	Years2	APC2	Years3	APC3	Years4	APC4	AAPC
**The Republic of South Africa**														
Stomach	1993-2015	-2.8*			-2.8*	1993-1998	2.2	1998-2015	-3.6*					-3.6*
Colorectum	1993-2015	1.2*			1.2*	1993-2015	-0.3							-0.3
Pancreas	1993-2015	0.6*			0.6*	1993-2003	1.9*	2003-2015	-0.6					-0.1
Lung	1993-2015	-0.8*			-0.8*	1993-2015	-0.1							-0.1
Breast	-	-	-	-	-	1993-2015	1*							1*
Uterus	-	-	-	-	-	1993-1996	7.5*	1996-2004	1.1*	2004-2013	-0.4	2013-2015	4.4	0.6
Ovary	-	-	-	-	-	1993-2009	2*	2009-2015	-1					0.8*
Prostate	1993-2015	2.3*			2.3*	-	-	-	-	-	-	-	-	-
Bladder	1993-2015	0.2			0.2	1993-2015	-1.1*							-1.1*
Leukemias	1993-2015	0.6*			0.6*	1993-2015	-0.2							-0.2
All cancers	1993-1999	2.6*	1999-2015	-0.5*	-0.5*	1993-1999	3.8*	1999-2015	-0.4*					-0.4*
**The Republic of Mauritius**														
Stomach	2001-2017	-4.1*			-4.1*	2001-2017	-4*							-4*
Colorectum	2001-2017	1.3*			1.3*	2001-2017	1.5*							1.5*
Pancreas	2001-2017	2*			2*	2001-2017	1.9							1.9
Lung	2001-2017	-1.7*			-1.7*	2001-2017	-1.4							-1.4
Breast	-	-	-	-	-	2001-2017	2.7*							2.7*
Uterus	-	-	-	-	-	2001-2017	-1.8*							-1.8*
Ovary	-	-	-	-	-	2001-2017	3.3*							3.3*
Prostate	2001-2017	-0.2			-0.2	-	-	-	-	-	-	-	-	-
Bladder	2001-2017	-1.1			-1.1	2001-2017	-0.3							-0.3
Leukemias	2001-2017	-0.3			-0.3	2001-2017	-1							-1
All cancers	2001-2017	-1*			-1*	2001-2017	0.1							0.1
**Réunion**														
Stomach	2001-2016	-4*			-4*	2001-2016	-3.1*							-3.1*
Colorectum	2001-2016	2.1*			2.1*	2001-2016	1.4							1.4

Cancer site	Men					Women								
	Years1	APC1	Years2	APC2	AAPC	Years1	APC1	Years2	APC2	Years3	APC3	Years4	APC4	AAPC
Pancreas	2001-2016	2.4*			2.4*	2001-2016	1.4							1.4
Lung	2001-2016	-1.3*			-1.3*	2001-2016	4*							4*
Breast	-	-	-	-	-	2001-2016	-0.1							-0.1
Uterus	-	-	-	-	-	2001-2016	-1.4							-1.4
Ovary	-	-	-	-	-	2001-2016	1							1
Prostate	2001-2016	-2.6*			-2.6*	-	-	-	-	-	-	-	-	-
Bladder	2001-2016	-2.6			-2.6	2001-2016	-0.2							-0.2
Leukemias	2001-2016	-3*			-3*	2001-2016	0.3							0.3
All cancers	2001-2016	-1.8*			-1.8*	2001-2016	-0.4							-0.4

APC: annual percent change. AAPC: average annual percent change. *Significantly different from 0 (p<0.05).

Supplementary Figure 1. Joinpoint analysis for mortality from major cancer sites in the Arab Republic of Egypt during the period 2000–2019.

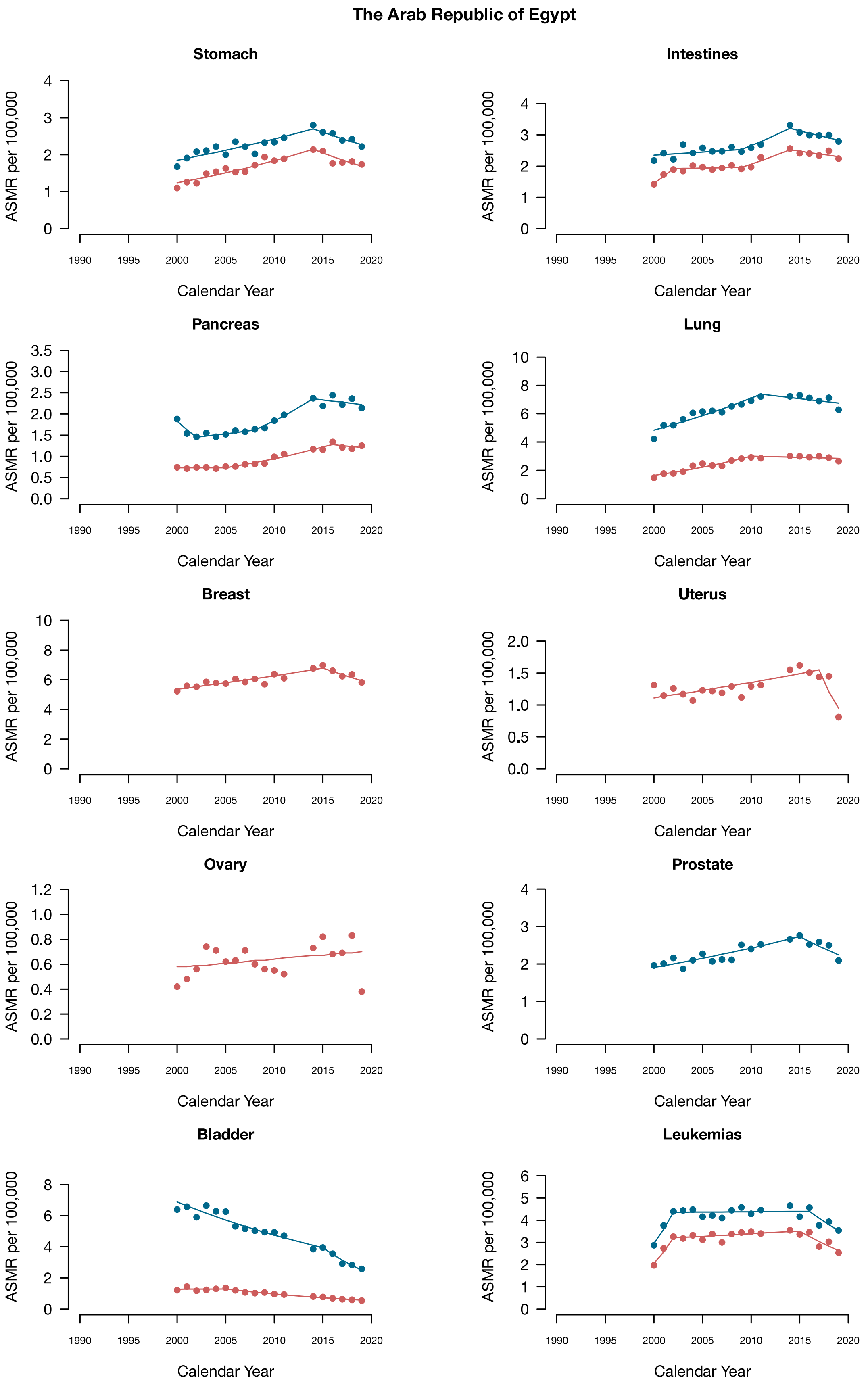

Supplementary Figure 2. Joinpoint analysis for mortality from major cancer sites in the Kingdom of Morocco during the period 2000–2016.

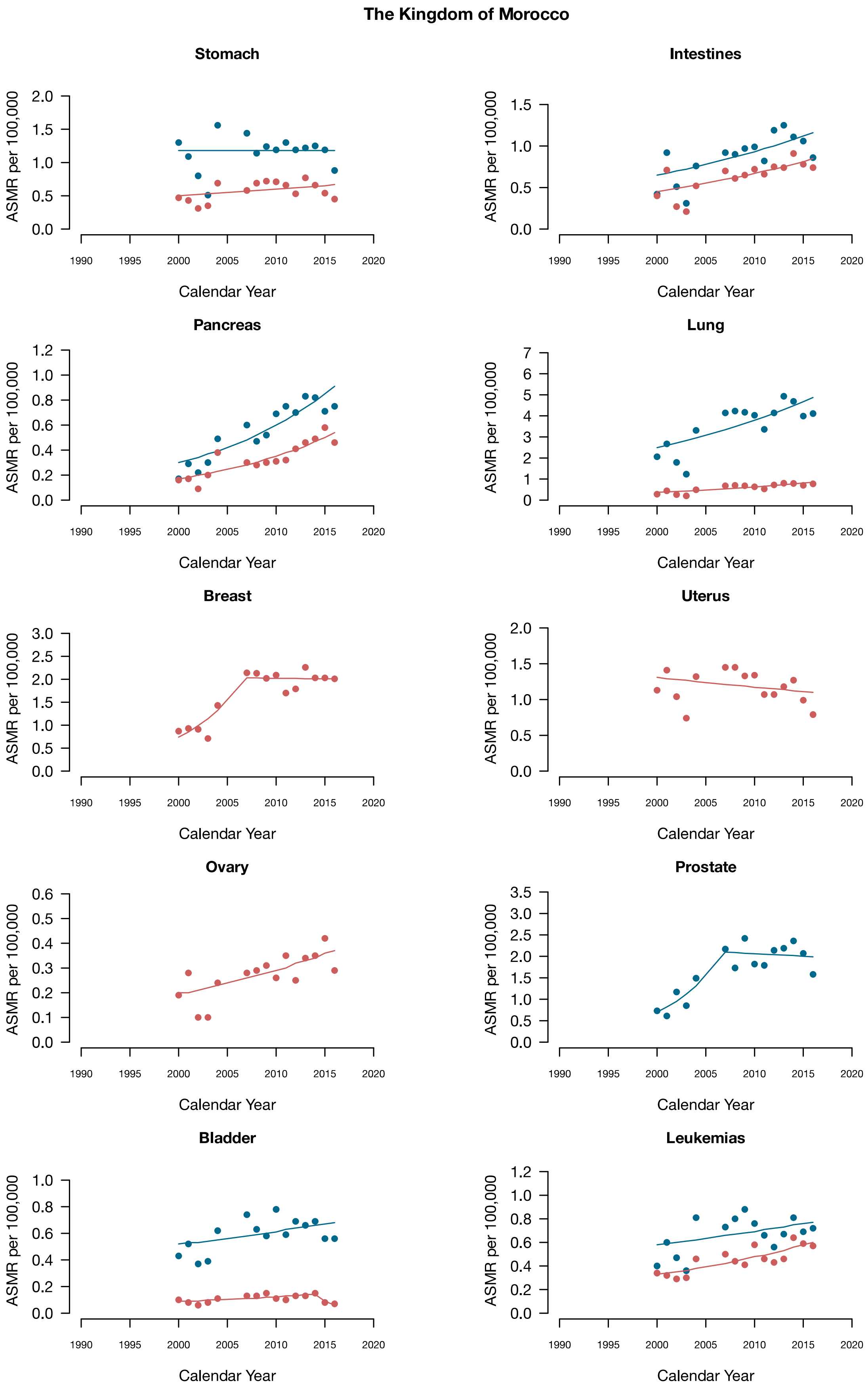

Supplementary Figure 3. Joinpoint analysis for mortality from major cancer sites in the Republic of South Africa during the period 1993–2015.

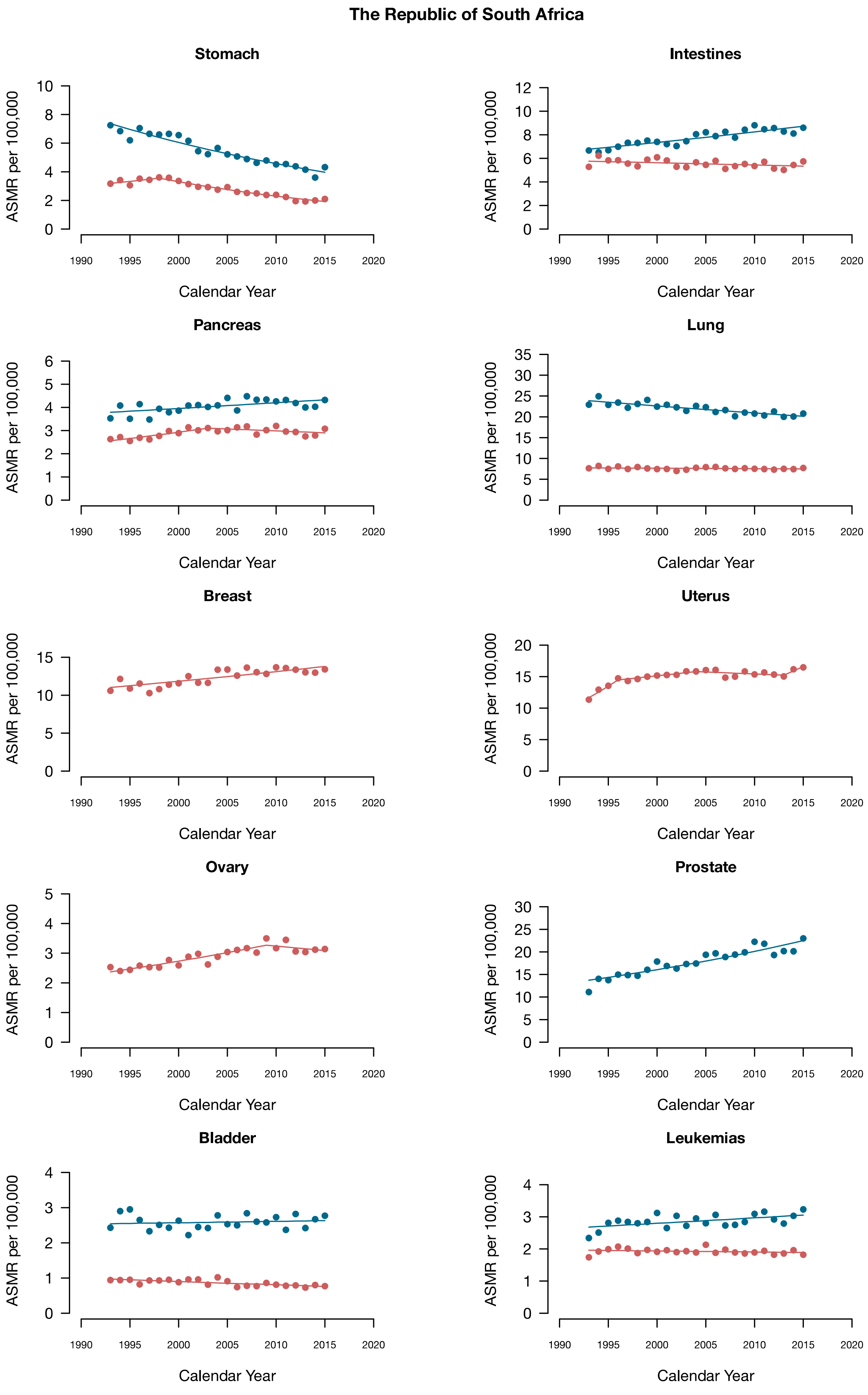

Supplementary Figure 4. Joinpoint analysis for mortality from major cancer sites in the Republic of Mauritius during the period 2001–2017.

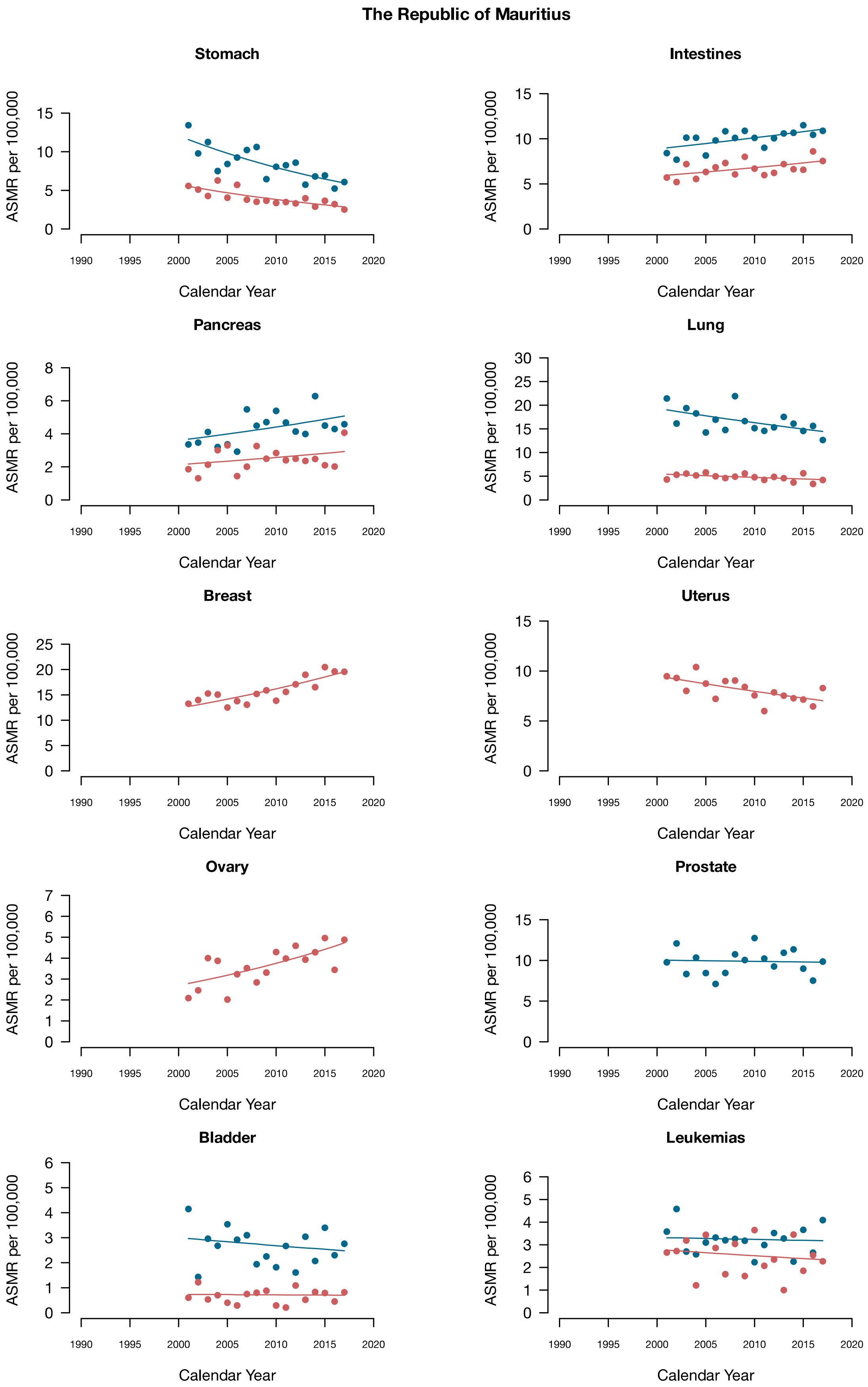

Supplementary Figure 5. Joinpoint analysis for mortality from major cancer sites in the Réunion during the period 2001–2016.

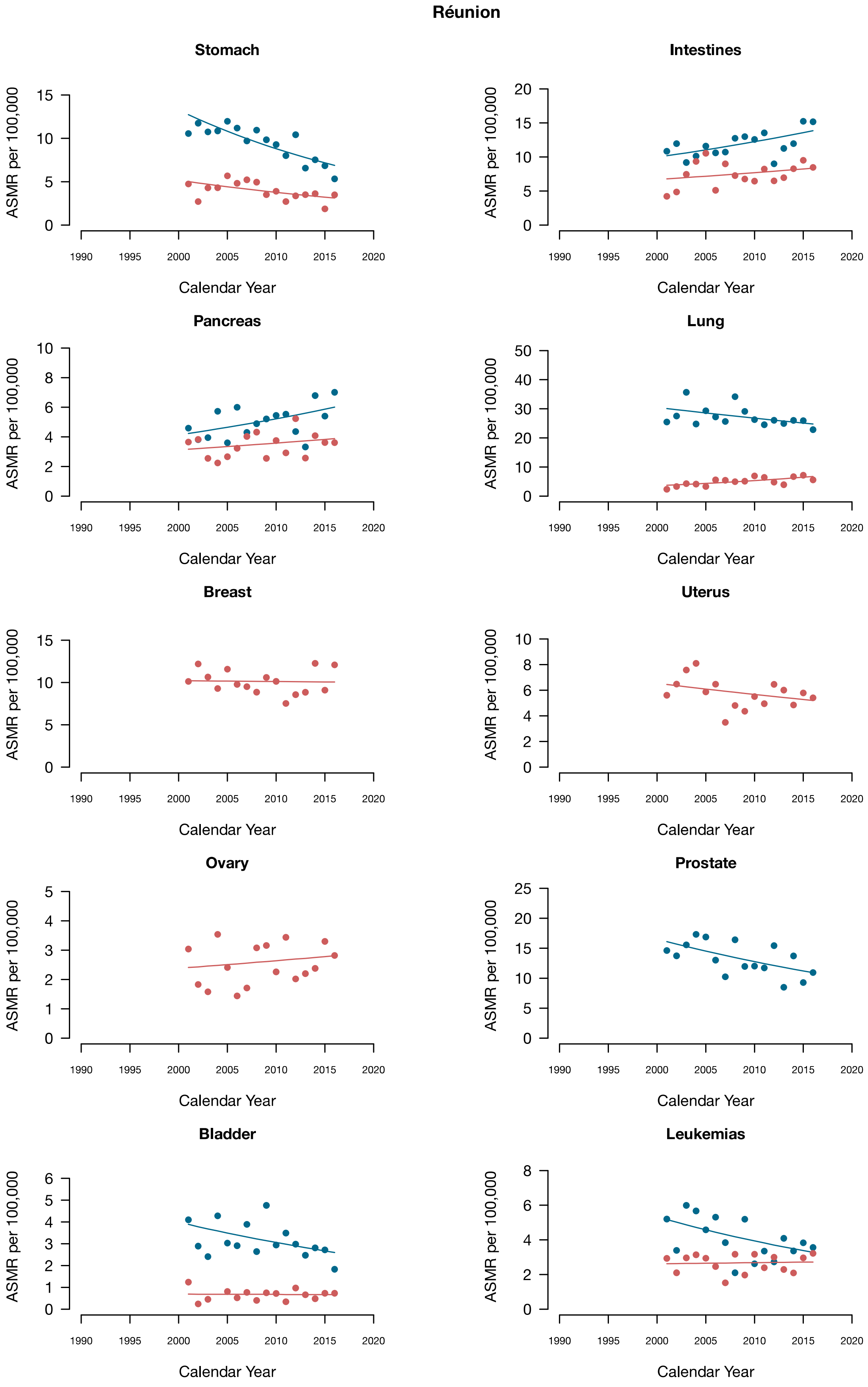

Supplement: Supplementary file 1 [file ejcp-33-192-s001.pdf]
